# Female adolescent elite handball players are more susceptible to shoulder problems than their male counterparts

**DOI:** 10.1007/s00167-018-4857-y

**Published:** 2018-02-10

**Authors:** Martin Asker, Lena W. Holm, Henrik Källberg, Markus Waldén, Eva Skillgate

**Affiliations:** 1 Musculoskeletal and Sports Injury Epidemiology Center, Institute of Environmental Medicine, Karolinska Institutet, Stockholm, Sweden; 2 Naprapathögskolan-Scandinavian College of Naprapathic Manual Medicine, Stockholm, Sweden; 3 Department of Monitoring and Evaluation, Public Health Agency of Sweden, Solna, Sweden; 4 Division of Community Medicine, Department of Medical and Health Sciences, Linköping University, Linköping, Sweden; 5 Department of Orthopaedics, Hässleholm-Kristianstad-Ystad Hospitals, Hässleholm, Sweden

**Keywords:** Athletic injuries, Prevalence, Overuse injury, Overhead sport, Throwing injury, Glenohumeral joint

## Abstract

**Purpose:**

Shoulder problems are frequent among senior elite handball players. The objective of this study was to assess the prevalence of shoulder problems among adolescent elite handball players and to investigate potential differences in gender, school grade, playing position and playing level.

**Methods:**

During the 2014 and 2015 pre-season periods, 471 players (age 15–18 years, 54% female) completed a comprehensive baseline questionnaire regarding history of any shoulder pain and shoulder problems experienced during the past season. The players were monitored weekly for one competition season (September–April) regarding shoulder problems and the amount of match and training. Generalised linear models with a binomial link function were used to calculate a prevalence ratio (PR) with 95% confidence interval (CI) to compare the subgroups of players.

**Results:**

In total, 110 players (23%) reported having substantial shoulder problems (defined as moderate/severe reduction in training volume, or moderate/severe reduction in performance, or complete inability to participate) at some point during the follow-up season, of which almost half reported complete inability to participate. Of those players reporting substantial problems, 43% (95% CI 39–48) did so for at least 3 consecutive weeks during the season. The prevalence was significantly higher in female players (PR 1.46, 95% 1.04–2.06) and in backcourt players (PR 1.58, 95% CI 1.08–2.32), but no differences were found for school grade (PR 1.21 95% CI 0.88–1.67) or playing level (PR 1.09 95% CI 0.76–1.56).

**Conclusions:**

The prevalence of substantial shoulder problems in adolescent elite handball players is high, especially among females, and this warrants further studies on risk factors for shoulder injury and the development of prevention strategies in handball players already before the age of 15. These findings also highlight the importance of introducing a clinical monitoring programme on a routine basis and improving the medical support, taking gender-related aspects into consideration, at handball-profiled secondary schools.

**Level of evidence:**

II.

## Introduction

Handball is a physically challenging sport that includes throwing, jumping, running and side-cutting movements as well as direct and indirect contact with opponents, which leads to a high injury rate [5]. The injury rate in youth handball players has been shown to be between 9.9 and 41.0 injuries per 1000 match hours and between 0.9 and 2.6 injuries per 1000 training hours [15, 19, 24]. Focusing on the incidence of newly sustained injuries exclusively leads, however, to the inability to describe the whole spectrum of overuse-related and long-standing shoulder problems among handball players [6, 8]. Recently, high prevalence of shoulder problems has been reported in both senior male and female elite players [1, 6, 16]. However, there is no study that has investigated the prevalence of shoulder problems among adolescent elite handball players, including potential associations with gender, school grade, playing position and playing level. The objective of this study was therefore to assess the prevalence of shoulder problems, and especially substantial problems, in adolescent elite handball players and to investigate potential differences in gender, school grade, playing position, and playing level. The hypothesis was that there is a higher prevalence of substantial shoulder problems in female players, in higher school grades, in backcourt players and in those competing at the highest playing level.

## Materials and methods

This study was based on data from a prospective cohort study called the Karolinska Handball Study (KHAST) with the main aim to investigate potential risk factors for developing shoulder injuries among male and female adolescent elite handball players in Sweden. Details about the general methodology, study population and the study design are reported elsewhere and will not be fully repeated below [3].

### Population

Briefly, ten out of 38 handball-profiled secondary schools in Sweden met the inclusion criteria and all these accepted to participate in the study [3]. Out of 552 eligible students aged 15–19 years in the ten schools, 471 male and female players were included during the 2014 and 2015 pre-season periods (Table 1).

**Table 1:** Characteristics of the 471 adolescent elite players in the study

	Females *n* = 256 (54%)	Males *n* = 215 (46%)
Age year, mean (SD)	16.4 (0.8)	16.4 (0.9)
Height cm, mean (SD)^a^	170.0 (9.2)	183.7 (6.7)
Weight kg, mean (SD)^a^	68.8 (8.6)	79.5 (11.1)
BMI, mean (SD)^a^	24.2 (8.5)	23.5 (2.8)
Years of playing handball, mean (SD)	9.2 (2.1)	9.0 (2.3)
School grade		
1st year students, *n* (%)	148 (58)	125 (58)
2nd year students, *n* (%)	69 (27)	59 (27)
3rd year students, *n* (%)	39 (15)	31 (15)
Playing position		
Goalkeepers, *n* (%)	35 (14)	37 (17)
Wing players, *n* (%)	45 (18)	50 (23)
Line players, *n* (%)	39 (15)	24 (11)
Backcourt players, *n* (%)	137 (53)	104 (49)
Level		
National level, *n* (%)	64 (25)	55 (26)
Regional level, *n* (%)	192 (75)	160 (74)

*BMI* body mass index, *SD* standard deviation

^a^Players who were available at the screening days (*n* = 442)

### Baseline questionnaire and weekly monitoring of shoulder problems

At inclusion, all players completed a baseline questionnaire based on Fahlström’s questionnaire [11], and a modified Swedish version of the Oslo Sports Trauma Research Center (OSTRC) overuse injury questionnaire [8, 10]. The OSTRC overuse injury questionnaire was modified in that instead of asking for shoulder problems during the past week, shoulder problems during the past season was asked for in the baseline questionnaire [3]. The players were also asked if they have had any previous shoulder pain during handball participation at any point during their carrier. The baseline questionnaire also included questions on playing position, match and training history, and playing level.

Players were thereafter monitored weekly during one competition season (September–April) during the 2014–2015 or 2015–2016 seasons. Shoulder problems, traumatic injuries, and match and training load were registered using a Swedish version of the OSTRC overuse injury questionnaire [10]. Briefly, an e-mail with the link to the questionnaire was sent to the players every Sunday morning. If the players did not respond initially, they automatically received an email reminder on a daily basis. If no response was received from the players by the following Wednesday, they also received a reminder via a short message service (SMS) including the link to the weekly report. If players also failed to respond to this SMS reminder within 2 days, a research assistant contacted them over the telephone. The survey software prevented incomplete reports by not allowing submission if one or more response were omitted.

If a player reported a traumatic injury, regardless of anatomical site, he/she was contacted by telephone within 2 days by one of two experienced sports medicine clinicians, for additional standardised questions regarding the anatomical site, injury situation, any medical care, and any injury diagnosis by a health care provider. If the player did not answer the phone call he/she received the questions about the traumatic injury by e-mail.

### Operational definitions

Based on information from the baseline questionnaire (regarding the preceding season) and from the weekly monitoring (regarding the follow-up season), shoulder problems were categorised into two types; any shoulder problems and substantial shoulder problems. If a player reported a problem in any of the four questions in the modified Swedish OSTRC overuse injury questionnaire this was categorised as any shoulder problem. For substantial shoulder problems, we used the same definition as Clarsen et al. “Players who reported (shoulder) problems leading to moderate or severe reductions in training volume, or moderate or severe reductions in sports performance, or complete inability to participate in sport” [8]. Consequently, players who selected options 3, 4 or 5 in questions 2 and/or 3 in the questionnaire were categorised as having substantial shoulder problems.

When comparing the prevalence between playing positions, wing and line players were categorised together as 6-m players. Playing level was dichotomised in national level, defined as players who played for an adolescent national team or were summoned to a national camp during the preceding season, and regional level, defined as players who did not have this exposure.

### Ethics

This study was approved by the Regional Ethics Review Board of the Karolinska Institutet, Sweden (2013/1722-31/4). All participating players, and legal guardians when appropriate, gave written informed consent before entering the study.

### Statistical analyses

Descriptive data, about player characteristics, are presented as numbers and mean values with standard deviation (SD) or proportions with 95% confidence interval (95% CI). Four types of prevalence measures were calculated: (1) week prevalence of any shoulder problems and substantial shoulder problems (defined as any shoulder problems or substantial shoulder problems during the preceding week during the follow-up season), (2) season prevalence of any shoulder problems and substantial shoulder problems retrospectively measured via the baseline questionnaire (defined as any shoulder problems or substantial shoulder problems during the preceding season), (3) season prevalence of any shoulder problems and substantial shoulder problems prospectively measured via the weekly reports (defined as any shoulder problems or substantial shoulder problems during the follow-up season), and (4) the lifetime prevalence of shoulder pain (defined as any shoulder pain during handball participation at some point during the handball carrier). The average weekly prevalence was calculated by dividing the number of players who reported having any shoulder problem and substantial problems, respectively, at each week during the season by the number of reports for that week [8]. The season prevalence was calculated by dividing the numbers of players who reported having any shoulder problem and substantial shoulder problems, respectively, at some point during the season by the total numbers of players in the cohort. Finally, the lifetime prevalence of shoulder pain was calculated by dividing the number of players who reported having had any previous shoulder pain during handball participation by the total numbers of players in the cohort.

To estimate the association between the different prevalence measures of shoulder problems/pain and gender, school grade, playing position, and playing level, prevalence ratio (PR) with 95% confidence intervals CI was calculated using generalised linear models with a binomial link function. All factors (gender, school grade playing position and playing level) were included in each model, respectively. Based on previous reported prevalence for shoulder problems from senior elite handball players [6, 16], and an estimation of the proportion of gender, school grade, playing position and playing level, using a power of 80%, a significance level of 5%, a drop-out rate of 10%, approximately 400 players were needed to describe the prevalence and PR, with a follow-up period of one competitive season.

Potential differences in gender, school grade, playing position and playing level, height, weight, BMI, and years of playing handball between those responding to less than 50% of the weekly reports and those responding to 50% or more was assessed using unpaired *t* test and Chi-square test. STATA software (STATA/ICIC 14.1, StataCorp, Texas, USA) was used for all statistical analysis and the level of significance was set at 0.05.

## Results

**Fig. 1:**
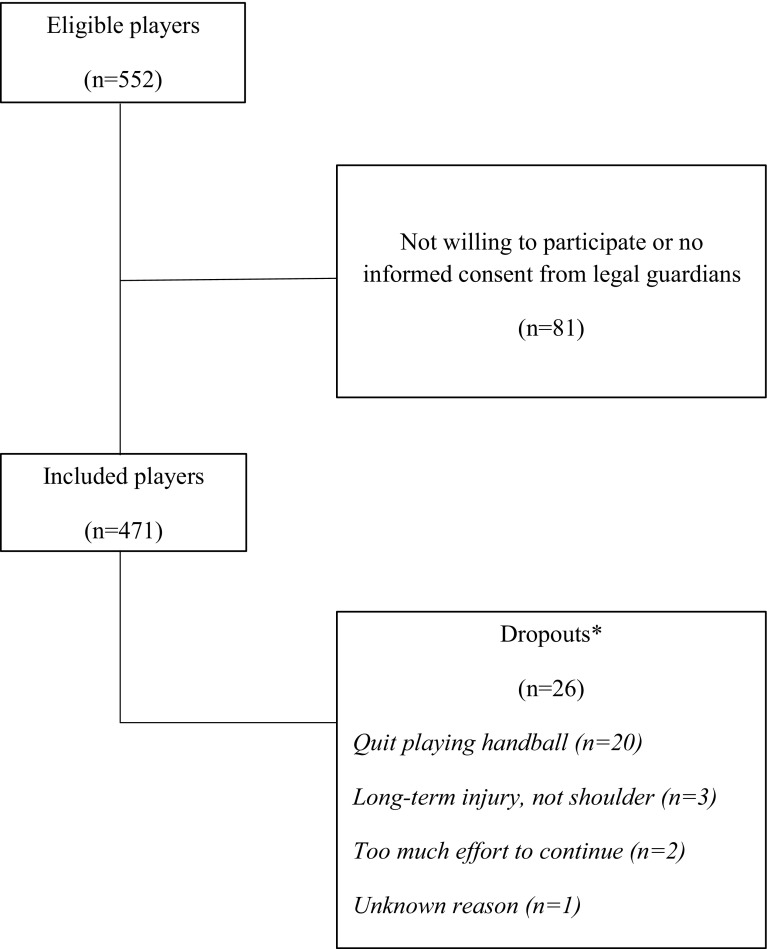
Study flow chart showing the recruitment, dropout and the response rate to the weekly reports. *Dropouts were included in the analysis and contributed as long they were in the study

In total, 81 players did not consent to participate in the study and another 26 players did not finish the follow-up, mainly due to quitting their studies at the handball-profiled school (Fig. 1).

The average response rate to the weekly questionnaire was 93% (range 87–98%), 73% of the players responded to all of the weekly reports and 85% of the players responded to more than 90% of the weekly reports. In total, 12,931 weekly reports were collected during the follow-up season.

### Prevalence of shoulder problems and pain

#### Week prevalence

The weekly prevalence of any shoulder problems and substantial shoulder problems, respectively, was 25% (95% CI 23–27) and 6% (95% CI 5–7).

#### Season prevalence during the preceding season

The prevalence of any shoulder problems and substantial shoulder problems during the preceding season (assessed retrospectively in the baseline questionnaire) was 28% (95% CI 24–32) and 13% (95% CI 10–16), respectively. The prevalence of any shoulder problems was higher in female players and in backcourt players (Table 2). The prevalence of substantial shoulder problems was higher in 2nd and 3rd grade students compared with 1st grade students (Table 2).

**Table 2:** Prevalence and prevalence ratios of any shoulder problems and substantial shoulder problems at some point during the preceding season

	No. of players	Any shoulder problems, *n* (%)	PR^a^ (95% CI)	Substantial shoulder problems, *n* (%)	PR^a^ (95% CI)
Total	471	133 (28)		60 (13)	
Gender					
Males	215	50 (23)	1.0	22 (10)	1.0
Females	256	83 (32)	1.36 (1.02–1.83)	38 (15)	1.36 (0.84–2.19)
School grade					
1st year students	273	70 (26)	1.0	21 (8)	1.0
2nd and 3rd year students	198	63 (32)	1.22 (0.92–1.62)	39 (20)	2.54 (1.55–4.17)
Playing position					
6-m players^b^	158	35 (22)	1.0	19 (12)	1.0
Backcourt players	241	85 (35)	1.59 (1.14–2.22)	38 (16)	1.33 (0.80–2.19)
Goalkeepers	72	13 (18)	0.82 (0.47–1.46)	3 (4)	0.36 (0.11–1.16)
Playing level					
Regional	352	100 (28)	1.0	45 (13)	1.0
National	119	33 (28)	0.98 (0.71–1.36)	15 (13)	1.04 (0.61–1.77)

*CI* confidence interval, *PR* prevalence ratio

^a^Adjusted for each other; gender, playing position, school grade and playing level

^b^6-m players include wing and line players

#### Season prevalence during the follow-up season

The season prevalence of any shoulder problems and substantial shoulder problems (assessed prospectively in the weekly reports) was 44% (95% CI 40–48) and 23% (95% CI 20–27), respectively. Of those reported substantial shoulder problems, 48% reported complete inability to participate due to shoulder problems. The prevalence of any and substantial shoulder problems were both higher in female players and backcourt players (Table 3).

**Table 3:** Prevalence and prevalence ratios of any shoulder problems and substantial shoulder problems at some point during the follow-up season

	No. of players	Any shoulder problems, *n* (%)	PR^a^ (95% CI)	Substantial shoulder problems, *n* (%)	PR^a^ (95% CI)
Total	471	207 (44)		110 (23)	
Gender					
Males	215	83 (39)	1.0	40 (19)	1.0
Females	256	124 (48)	1.25 (1.02–1.54)	70 (27)	1.46 (1.04–2.06)
School grade					
1st year students	273	116 (42)	1.0	59 (22)	1.0
2nd and 3rd year students	198	91 (46)	1.11 (0.91–1.35)	51 (26)	1.21 (0.88–1.67)
Playing position					
6-m players^b^	158	62 (40)	1.0	29 (18)	1.0
Backcourt players	241	124 (51)	1.32 (1.05–1.66)	70 (29)	1.58 (1.08–2.32)
Goalkeepers	72	21 (29)	0.75 (0.50–1.12)	11 (15)	0.84 (0.45–1.59)
Playing level					
Regional	352	155 (44)	1.0	80 (23)	1.0
National	119	52 (44)	0.99 (0.79–1.24)	30 (25)	1.09 (0.76–1.56)

*CI* confidence interval, *PR* prevalence ratio

^a^Adjusted for each other; gender, playing position, school grade and playing level

^b^6-m players include wing and line players

#### Lifetime prevalence of shoulder pain

The lifetime prevalence of shoulder pain (assessed retrospectively in the baseline questionnaire) was 41% (95% CI 36–45), and was higher among female players compared with males, 46 versus 35% (PR 1.26, 95% 1.01–1.57) and among backcourt players compared with 6-m players, 51 versus 32% (PR 1.57, 95% CI 1.22–2.03). The lifetime prevalence of shoulder pain was also higher among 2nd and 3rd grade students compared with 1st grade students, 45 versus 37% (PR 1.22, 95% CI 0.99–1.50). No difference was seen for playing level, 43 versus 41% (PR 1.07, 95% CI 0.84–1.35).

### Duration of the shoulder problems

In total, 75% (95% CI 69–81) of those with any shoulder problems reported such problems for at least 3 consecutive weeks during the season. Similarly, 43% (95% CI 39–48) of those with substantial shoulder problems reported such problems for at least 3 consecutive weeks during the season. Of those with shoulder problems and substantial shoulder problems at some point during the preceding season, 77% (95% CI 66–85) and 67% (95% CI 58–75) also reported such problems at least once during the follow-up season.

### Comparison between players with low and high response rates

There were no significant differences between those responding to less than 50% of the weekly reports (*n* = 33) and those responding 50% or more (*n* = 438) in gender, school grade, playing position, and playing level as well as in height, weight, BMI, and years of playing handball (ns).

## Discussion

This is the first study to describe the association between shoulder problems and gender, school grade, playing position, and playing level in adolescent elite handball players. The principal findings were a high prevalence of shoulder problems, where almost one in four players reported having substantial shoulder problems at some point during a handball season, and that the shoulder problems were significantly more prevalent among female players and backcourt players.

### Prevalence of shoulder problems

Describing the prevalence and burden of injuries is an important first step to develop injury prevention strategies [22]. The average weekly prevalence of substantial shoulder was 6% in this study, which is similar to figures reported in recent studies on senior handball players in Norway [1, 6, 8]. Clarsen et al. [6] reported that 24% of the players had substantial shoulder problems in their dominant shoulder at some point during the season with an average weekly prevalence of 12%, while Andersson et al. [1] and Clarsen et al. [8] reported average weekly prevalence of 8 and 6%, respectively. Our results are also similar to the weekly average prevalence of 5% recently reported in Norwegian secondary school volleyball players [7]. Furthermore, 46% of the female players and 35% of the male players in our study reported having a history of previous shoulder pain, and this is in line with a study in which a lifetime prevalence of shoulder pain was 44% in senior Iranian male and female handball players [14]. Our results are, however, somewhat lower than what has been reported in Norwegian senior elite female handball players, where it was reported that 57% were affected by previous or current shoulder pain [16].

### Gender

Female players had a significantly higher prevalence of both any shoulder problems and substantial shoulder problems during the follow-up period. These results are in line with the finding from a recent study on Norwegian senior elite handball players where a higher season prevalence of shoulder problems was reported in female players (26%) compared to male players (20%) [2]. A similar gender-related difference was also reported in Brazilian senior elite players where 9% of the female players reported traumatic shoulder injuries compared to 3% of the male players [12]. Interestingly, similar gender-related differences, with higher rates identified for females, have also been identified for other injuries in team sports such as concussion [18], and anterior cruciate ligament (ACL) injury [17, 23]. However, a recent study on youth handball players did not find any gender difference in shoulder injury incidence [15] and Giroto et al. [12] reported no differences in the proportion of reported overuse shoulder injuries in Brazilian elite senior players. Importantly, there were a higher proportion of female players who reported previous shoulder pain at baseline in our study. Female players may therefore develop shoulder problems at a younger age than do male players, which also would be similar to the relative age effect identified in the ACL injury discrepancy [23]. In addition, female players have demonstrated a higher relative workload compared with male players [4], and this could influence the risk of developing shoulder problems. Finally, Serrien et al. [20] showed that the throwing kinematics differed between male and female players, where male players showed a higher endo/ecorotation in trunk velocity and higher shoulder horizontal abduction angles during the cocking phase. Also, van Den Tillaar et al. showed that the maximal endpoint velocities of the hand and wrist segment during throwing were higher in male players, [21] but no other major gender differences in kinematics were found. Gender-related differences in throwing kinematics may thus influence the risk of developing shoulder problems in handball players, but there is currently no study that investigated this.

### School grade

There was no significant difference in the prevalence of any or substantial shoulder problems between 1st grade students and 2nd and 3rd year students during the follow-up season in our study. Interestingly, however, the prevalence of substantial shoulder problems during the preceding season was 8% in the 1st grade students compared with 20% in the 2nd and 3rd grade students. The higher prevalence among 2nd and 3rd grade players could possibly be explained by the fact that most of the players still reported substantial shoulder problem the following season. Another plausible explanation would be that both the physical and psychological demands on the players increase when starting a handball-profiled secondary school. These findings are contrary to the findings in a recent study by Moller et al. [15] where there was no difference in occurrence of shoulder injuries between under-16 and under-18 male and female handball players. However, it should be noted that only time-loss injuries were reported in that study and they used a different injury classification, which might explain the discrepancy between the two studies.

### Playing position

Backcourt players had a significantly higher prevalence compared with wing and line players in all categories of shoulder problems in this study. This finding is perhaps not surprising since a backcourt player has a well-known higher overall demand on the shoulder, with largely more frequent high-velocity throwing and they also have a higher exposure to getting stopped by an opponent during breakthroughs or blocking opponent shots which puts the shoulder in vulnerable positions [13].

### Playing level

There was no significant differences in the prevalence of shoulder problems between players competing at a national level during the preceding season and those playing only at a regional level, regardless of which definition we used. The most likely explanation to this finding is that all players in our study played handball at a high level, which is a strict criterion for being accepted to study at a handball-profiled secondary school in Sweden. These results are also in line with those in a recent study by Clarsen et al. [6] who showed no association between playing level and shoulder injuries in senior male handball players in Norway.

### Methodological considerations

The major strength with this study is the large sample size (471 players) and that the sample is also most likely to be representative for the total population of adolescent elite handball players in Sweden. Also, and in contrast to many previous studies describing shoulder problems in overhead athletes [6, 9, 25, 26], this study has a large number of girls and boys, which thus enables to investigate potential gender-related differences with respect to prevalence of shoulder problems. Moreover, female and backcourt players had significant higher prevalence of shoulder problems regardless of what definition used which strengthens the validity of the results. Finally, to reduce the risk of selection bias, a great effort was put on obtaining a high response rate in this study. To our knowledge, the average weekly response rate of 93% during a season is among the highest ones ever reported in studies on sports injuries using weekly questionnaire data distributed by email/SMS.

Some limitations of this study should also be noted. First, the number of missed days, training sessions or matches or specific shoulder diagnoses was not recorded, since the methodology for self-reporting of complaints according to the OSTRC model was strictly used [8]. Second there could be a risk of misclassification of the outcome when reporting any previous shoulder pain or shoulder problems during the preceding season because the questionnaire used was originally designed to report shoulder problems during the past week. Together with a possible recall bias this may therefore explain why the prevalence reported for the preceding season was lower compared with the follow-up season. Third, this study might be underpowered for analysing differences between school grades, thus these aspects should be addressed further in future studies. Last, approximately every seventh eligible player did not consent to participate in the study, which was slightly higher than expected. As in all studies assessing the prevalence of a condition, there is always a risk of selection bias that may lead to either an under- or an over-estimation of the prevalence. However, the main reason for not being included in this study was that players could not get the written consent from their legal guardians in time before data collection was initiated.

## Conclusion

The prevalence of substantial shoulder problems in adolescent elite handball players is high, especially among females, and this warrants further studies on risk factors for shoulder injury and the development of prevention strategies in handball players already before the age of 15. These findings also highlight the importance of introducing a clinical monitoring programme on a routine basis and improving the medical support, taking gender-related aspects into consideration, at handball-profiled secondary schools.
